# FGF/FGFR signaling pathway involved resistance in various cancer types

**DOI:** 10.7150/jca.40531

**Published:** 2020-02-03

**Authors:** Yangyang Zhou, Chengyu Wu, Guangrong Lu, Zijing Hu, Qiuxiang Chen, Xiaojing Du

**Affiliations:** 1 Department of Rheumatology and Immunology, the Second Affiliated Hospital and Yuying Children's Hospital of Wenzhou Medical University, Wenzhou, Zhejiang 325000, China; 2 Department of Gastroenterology, The Second Affiliated Hospital and Yuying Children's Hospital of Wenzhou Medical, Wenzhou, Zhejiang 325000, China); 3 College of Pharmaceutical Sciences, Wenzhou Medical University, Wenzhou, Zhejiang 325000, China; 4 Department of Ultrasonic Imaging, the First Affiliated Hospital of Wenzhou Medical University, Wenzhou, Zhejiang 325035, China; 5 Department of Gastroenterology, the First Affiliated Hospital of Wenzhou Medical University, Wenzhou, Zhejiang 325035, China

**Keywords:** FGF/FGFR, resistance, oncotherapy, FGF/FGFR inhibitors

## Abstract

Resistance becomes major clinical issue in cancer treatment, which strongly limits patients to benefit from oncotherapy. Growing evidences have been indicative of the critical role of fibroblast growth factor (FGF)/receptor (FGFR) signaling played in resistance to oncotherapy. In this review we discussed the underlying mechanisms of FGF/FGFR signaling mediated resistance to chemotherapy, radiotherapy and target therapy in various cancers. Meanwhile, we summarized the reported mechanism of FGF/FGFR inhibitors resistance in cancers.

## Introduction

Fibroblast growth factor (FGF) family being part of the receptor tyrosine kinase (RTK) families are comprised of 18 ligands, exerting their functions through four highly conserved tyrosine kinase receptors (including FGFR1, FGFR2, FGFR3 or FGFR4) 1, 2. FGF/FGFR signaling controlled multiple physiological processes, such as endocrine homeostasis, wound repair, and cellular behaviors (including proliferation, differentiation and survival), via activation of fibroblast growth factor receptor substrate2 (FRS2), mitogen activated protein kinase (MAPK)/extracellular signal-regulated kinase 1/2 (ERK1/2), phosphoinositide 3-kinase (PI3K)/protein kinase B (AKT) signaling pathways, signal transducer and activator of transcription3 (STAT3), phospholipase Cγ (PLCγ) and ribosomal protein S6 kinase 2 (RSK2) 1,2. These characteristics make FGF/FGFR signaling impressionable to be hijacked by oncocytes. The underlying mechanism driving FGF/FGFR signaling is very cancer special, and can be classified into FGFR amplification 3, FGFR mutation 4, abnormity of FGFR involved ligands 5 and nuclear translocation of FGF/FGFR 6. Recently, increasing studies indicated the close connection of FGF/FGFR signaling with the resistance to oncotherapy in various cancer types. These studies make FGF/FGFR to be a highly potential therapy target in cancers, and drugs targeting FGF/FGFR have been a meaningful and hopeful field in anti-tumor treatment study. However, as a target therapy, scholars are also starting to pay attention to the resistance to FGF/FGFR inhibitors in cancers. In the paper, we reviewed the FGF/FGFR signaling pathway involved resistance in cancer treatment below.

## FGF/FGFR signaling pathway mediated resistance to treatment in various cancers

**Figure 1:**
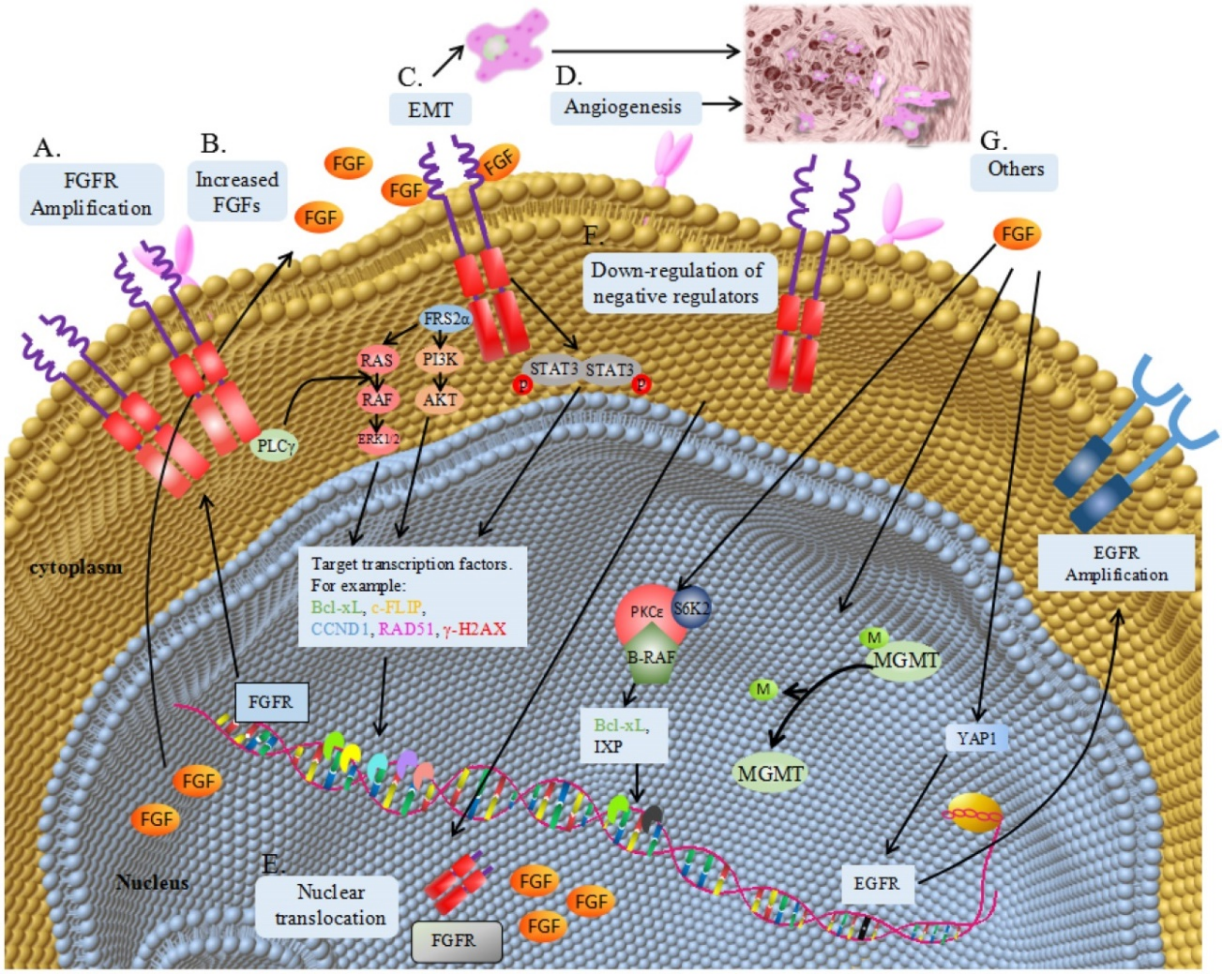
** Mechanism of FGF/FGFR signaling pathway involved in resistance to oncotherapy. (A)** Gene amplification translates into FGFR overexpression, causing receptor accumulation and continuously activation of the down-stream signaling pathways, including ligands-dependent and independent pathways. **(B)** Increased FGF from tumor cell or microenvironment can overstimulate FGFR and its down-stream signaling pathways. **(C)** FGF/FGFR signalling may contribute to epithelial-mesenchymal transition (EMT). **(D)** FGF/FGFR signalling can promote angiogenesis and further induce resistance to antiangiotherapy. **(E)** Nuclear translocation of FGF or FGFR can accelerate DNA repairor regulate gene transcription. **(F)** Downregulation of negative regulated proteins, such as SPRY, can maintain the activation of FGF/FGFR signalling. **(G)** Activation of other survival signalling. FGF can regulate the expression of Bcl-xL and IXP via a protein complex containing S6K2, PKCɛand B-Raf. FGF can promote the demethylation of O^6^-methylguanine-DNA-methyltransferase (MGMT) promoter. FGF can upregulate the expression of EGFR via an intermediate protein YAP1.

The underlying mechanism of FGF/FGFR signaling pathway involved in resistance to oncotherapy can be generally divided into over-expression of ligands or receptors, epithelial-mesenchymal transformation (EMT), angiogenesis, nuclear translocation, downregulation of negative regulator and activation of other survival signaling (Table 1 and Figure 1).

### Lung cancer

Lung cancer, including two subclasses, non-small cell lung cancer (NSCLC) and small cell lung cancer (SCLC), is the first leading cause of malignant tumor death around the world 7. Epidermal growth factor receptor (EGFR) inhibitors, such as erlotinib, gefitinib and afatinib, achieved dramatic response in EGFR addicted NSCLC, while nearly all develop resistance finally 8. EMT is one confirmed molecular mechanism of resistance to EGFR inhibitors in NSCLC 9. It has been shown that mesenchymal NSCLC cells have a distinct reduction in sensitivity to EGFR inhibitors 10. These cells frequently exhibit aberrant expression of FGFR and related autocrine signaling, which can activate MAPK and PI3K pathways, further leading to the resistance to EGFR inhibitors 10. In turn, aberrant FGF or FGFR expression also drive EMT in NSCLC and subsequently induce resistance to EGFR inhibitors 11. Some papers revealed that EGFR inhibitors treatment can upregulate several FGFs/FGFRs, for instance, FGF2, FGF9, FGF13, FGFR1, FGFR2 and FGFR3 12-15. FGF/FGFR provide an autocrine receptor tyrosine kinase-driven bypass pathway and induce resistance via their conventional downstream cascades (ERK1/2, AKT and STAT3) in NSCLC, which are initially sensitive to EGFR inhibitors 12-15. KRAS mutations occur in about 15-30% of NSCLC and often correlate a poor response to EGFR inhibitors 16,17. Blockade of downstream factors of KRAS-driven pathways, such as MEK, becomes a preferable option for the therapy of this NSCLC cohort. However, MEK inhibitor, trametinib, induces a compensation referring to FGFR1, leading to signaling rebound and adaptive drug resistance 18. A combination of trametinib and FGFR inhibitors could be perceived as an effective strategy for treating KRAS mutant NSCLC 19. Cyclin-dependent kinase 4 (CDK4) has been emerging as another target in KRAS mutant NSCLC. Eric Haines et al indicated that FGFR1 triggered MAPK-mTOR pathway and decreased the sensitivity of NSCLC to CDK4 inhibitor, Palbociclib 20.

SCLC, accounting for 20% of lung cancer, could develop rapidly to chemo-resistance, harbouring a poor overall survival 21. FGF2 has been reported to be main culprit for this chemo-resistance 21,22. FGF2 post-transcriptionally modulates inhibitor of apoptosis protein (IAP) levels, decreasing the releasing of second mitochondria-derived activator of caspases (Smac) (progress induced cell apoptosis in response to cell stress), then restraining etoposide-induced cell apoptosis in SCLC cells 21. Another paper revealed the detailed molecule mechanism underlying how FGF2 upregulates the expression of anti-apoptotic proteins including IAP and Bcl-xL. FGF2 could induce the formation of a protein complex containing S6K2 (ribosomal protein S6 kinase 2), PKCɛ (protein kinase C epsilon) and B-Raf, which further directly increases the expression of IAP and Bcl-xL 22.

### Breast cancer

Estrogen receptor (ER) expresses in approximately 60% of breast cancer (BC). These ER positive BC patients initially respond well to antiestrogens like tamoxifen or Fulvestrant, while they gradually become resistant. Growth of FGF1 or FGF4 transfected ER positive BC cells is not inhibited by treatment of antiestrogen 23. The autocrine activity in FGF transfected BC cells may bypass the requirement for ER activation in tumor growth 23. Further study revealed that the antiestrogen resistance may be due to FGF1 induced activation of MAPK and PI3K pathway via FGFRs 24,25. Nicholas Turner et al found that FGFR1 amplification could drive both ligands dependent and independent signaling pathway, which promoted CCND1 (cyclin D1) expression in ER positive BC, resulting in the resistance to antiestrogen 26. Another paper demonstrated that FGF2 secreted from tumor microenvironment could also upregulate CCND1 expression and mediate resistance to antiestrogen 27. Moreover, FGFR1 could also transfer to cell nuclear and regulate the transcription of ER dependent target genes 28. Taken together, FGF/FGFR can take the place of ER and activate ER associated down-stream signaling in antiestrogen resistant BC cells, which may account for the resistance to antiestrogen.

Anti-vascular endothelial growth factor (VEGF) therapy has been a promising therapeutic option for cancers, while it failed to improve survival in BC patients 29. Data from mouse models indicated that diet-induced obesity weakens the effect of anti-VEGF therapy on BC 29. Upregulation of IL-6 or FGF-2 in obesity may account for the resistance to anti-VEGF therapy 29. Chemotherapy is also the treatment option for BC patients. It has been reported that FGF2 and FGFR4 were involved in the resistance to chemotherapy in BC 30. Upregulated FGFR4 reduced apoptosis sensitivity to doxorubicin or cyclophosphamide via decreaseing phospho-ERK1/2 levels and Bcl-xL expression in BC^30^. Min Xu et al, however, indicated that FGFR4 caused resistance to doxorubicin through increasing glucose metabolism in BC cells 31. In Shenduo Li et al's paper, nuclear FGF2 promoted resistance to doxorubicin via increasing DNA-dependent protein kinase (DNA-PK) expression, which could accelerate DNA repair 32.

### Colorectal cancer

Data from 172 rectal carcinoma specimens who underwent neoadjuvant concurrent chemoradiotherapy (CCRT) indicated that high-level expression of FGFR2 is positively associated with advanced stage tumor, poor treatment response and poor prognosis 33. A systems biology method revealed new genes and pathways involving in drug resistance to oxaliplatin and 5-fluorouracil (5-FU) in colorectal cancer (CRC) and presented FGFR4 as a potential agent of chemo-resistance 34. Synergistic interaction between FGFR4 silencing and 5-FU or oxaliplatin has been confirmed to have better anti-cancer activity in CRC cell lines 34. The research pointed out that anomalous expression of FGFR4 increased the activation of pro-survival STAT3 transcription factor and expression of cellular FLICE-like inhibitory protein (c-FLIP, an anti-apoptotic protein), leading to chemo-resistance in CRC 34. Overexpression of FGFR4 has also been reported in radio-resistant CRC 35. In CRC cells, enforced expression of FGFR4 stabilizes recombination protein A (RAD51, an important component of DNA double strand fracture repair) levels 36, leading to increasing clearance of γ-H2AX foci (an indication for DNA injury) 37 and cell survival in the mismatch repair-proficient cells 35.

### Gastrointestinal stromal tumor

Imatinib, selectively targeting KIT or PDGFRA, has observably improved prognosis in advanced gastrointestinal stromal tumors (GISTs) 38. However, about half of GISTs cases treated with imatinib gradually develop acquired resistance in the first two years 39. This event may attribute to secondary KIT mutations 39, while part patients become resistance to imatinib without KIT mutations. Aberrant FGF2 secretion has been detected in imatinib-resistant GISTs cell lines 40. Addition of FGF2 to GISTs cells reverted KIT phosphorylation during imatinib therapy and reduced the anti-tumor activity of imatinib in GISTs, while RNAi-mediated silencing of FGFR3 could cancel out this phenomenon 40. Further investigation indicated that FGFR3 crosstalk with KIT stimulated the MAPK pathway to facilitate resistance to imatinib 40. Another study indicated that continuous activation of KIT in GISTs cells depressed FGFR1 signaling through ERK-dependent feedback mediated by Sprouty (SPRY) proteins 41, a negative regulator of FGFR 42. Blockage of this feedback loop by imatinib enhanced FGFR1 activation and its down-stream signal pathway, resulting in the poor response of GISTs to imatinib 41. Indeed, combined treatment with FGFR inhibitors and imatinib can achieve anti-tumor activity in imatinib resistant GISTs 43,44.

### Squamous cell carcinoma

Increase of released FGF2 has been reported in the squamous cell carcinoma (SCC) cell lines after irradiation 45. The increased FGF2 decreased the sensitivity of SCC cells to the subsequent irradiation 45. Another paper indicated that significant upregulation of FGFR3 has been found in radio-resistant SCC cell lines 46. FGF2 or FGFR3 inhibition could provide a potent radio-therapeutic strategy for SCC 46. Data from a SCC xenograft model suggested that upregulation of FGF2 and FGFR3 has also been found in tumor cells with resistance to anti-VEGF therapy 47. The paper demonstrated that bevacizumab-refractory tumor cells converged on ERK signaling to upregulate FGF2, which mediated aversion of anti-VEGF therapy in turn 47. Shin Saito et al demonstrated that FGF/FGFR signaling also mediated the lapatinib resistance in esophageal SCC 48.

### Osteosarcoma

Previous study on 352 osteosarcoma patients, who received neo-adjuvant chemotherapy, indicated that amplification of FGFR1 gene was found exclusively in the poor response group and represented approximately 18.5% of patients in the group 49. Due to the comparatively low rate cases with FGFR1 amplification in the study, the essential connection between FGFR1 amplification and poor response to chemotherapy need to be further confirmed. Catarina R et al found that FGF2 promoted the resistance of osteosarcoma cells to cisplatin through a mechanism that involved PKCε, B-RAF and S6K2 protein complexes, which could further control the expression of several anti-apoptotic proteins 50.

### Hepatocellular carcinoma

FGF8 upregulated the expression of EGFR in hepatocellular carcinoma (HCC) via increasing the expression of yes-associated protein 1 (YAP1), which then facilitated the resistance to EGFR inhibitors 51. Victoria Tovar et al uncovered that the FGF/FGFR signaling enriched, including PI3K cascade, in the HCC cells after long-time exposure to sorafenib 52. Inhibition of FGF/FGFR signaling can also revert resistance in sorafenib-acquired resistant tumors 52,53.

### Pancreatic tumors

Antiangiogenic therapy could trigger upregulation of other pro-angiogenic factors in pancreatic tumors. FGF/FGFR signaling made great contribution to escaping anti-angiogenic targeting of VEGF signaling in pancreatic islet tumors and pancreatic neuroendocrine tumors 54,55. Combined inhibition of FGFR and VEGFR revert the adaptive resistance to VEGFR targeted therapies in pancreatic tumors 54,55.

### Melanoma

Bromodomain and extraterminal (BET) inhibitors could increase the expression of FGFR in uveal melanoma (UM) cells 56. FGF2 can rescue the UM cells from growth inhibition by BET inhibitors via FGFR 56. When liver metastasis occurred in UM, BET inhibitors would be ineffective *in vivo*, because hepatic stellate cells (HSCs) secreted FGF2 to promote the resistance to BET inhibitors 56. Methylation of the O^6^-methylguanine-DNA-methyltransferase (MGMT) promoter decreased the expression of its associated protein O^6^ -alkylguanine-DNA-alkyltransferase (AGT) 57. FGF2 could induce the demethylation of MGMT promoter and further increase the expression of AGT, which then promote the resistance of melanoma to temozolomide 57.

### Hematological malignancies

Overexpression of FGFR3 in myeloma confers resistance to dexamethasone, partly via upregulating of Bcl-xL 58. However, FGFR3 did not alter sensitivity of myeloma to melphalan or doxorubicin 58. Chronic lymphocytic leukemia (CLL) cells with high intracellular levels of FGF2 appeared to be more resistant to fludarabine treatment, while the underlying mechanism need to be further explored 59.

### Urinary tumors

FGF/MAPK signaling detours a requirement for androgens and the androgen receptor in promoting growth of prostate cancer 60. Cotargeting of FGF/MAPK signaling with capability of providing cell survival and proliferation signals could reverse castration resistance in prostate cancer 60. In urothelial cancer, FGFR inhibitor ASP5878 can effectively reverse the acquisition of gemcitabine or adriamycin resistance 61.

## Molecular mechanism of resistance to FGF/FGFR target therapy in cancers

**Figure 2:**
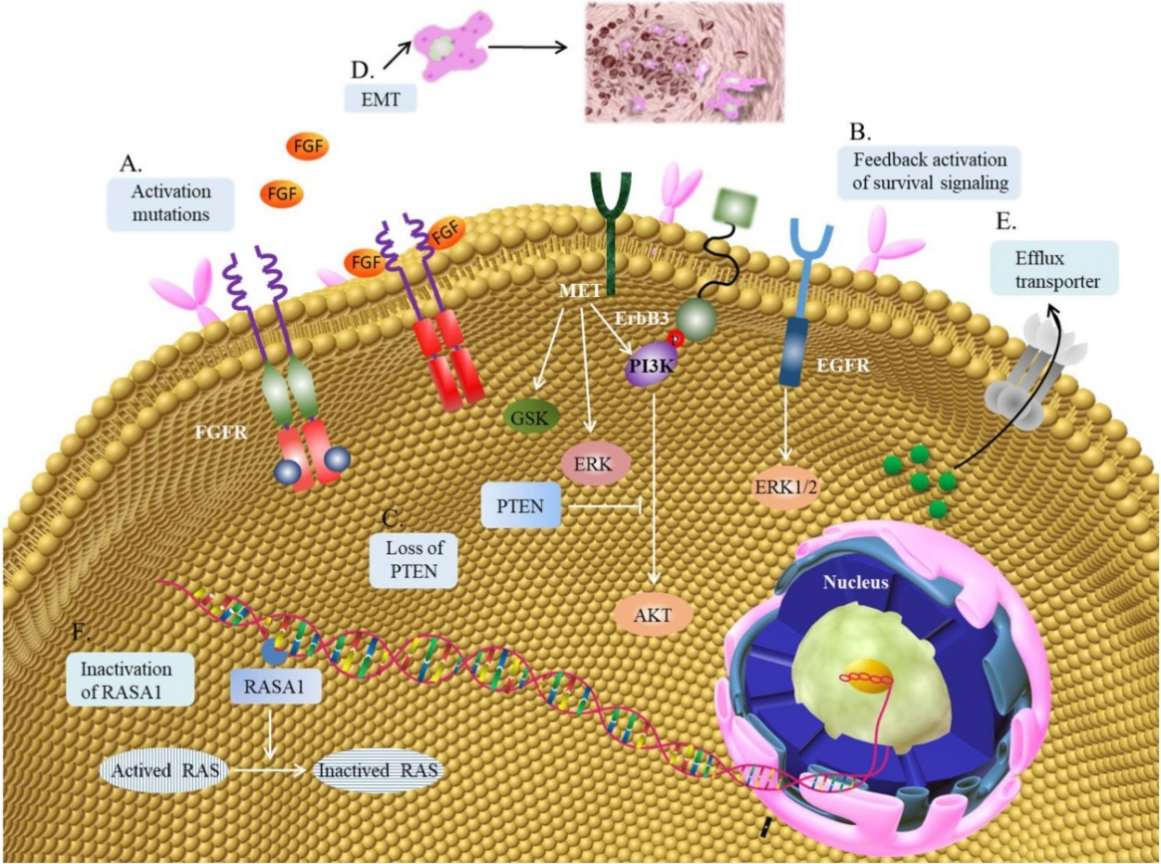
** Mechanism of resistance to FGF/FGFR inhibitors. (A)** Gatekeeper mutation in the FGFR kinase domain often results in resistance to FGFR inhibitors. **(B)** FGFR inhibition can activate other membrane receptor signaling pathwayin the feedback way, like EGFR, ERBB3 or MET. The downstream cascades may be mediated by MAPK-ERK1/2, PI3K-AKT and GSK signaling pathway. **(C)** Loss of PTEN can increase the activation of PI3K-AKTsignaling pathway. **(D)** EMT can contribute to the resistance to FGFR inhibitors. **(E)** ABCG2 regulated drug-efflux can also contribute to the resistance to FGFR inhibitors. **(F)** RASA1 is a protein activator, which can promote the translation of activated RAS to inactivated RAS. The inactivation of RASA1 may induce the resistance to FGFR inhibitors.

The contribution of abnormal FGF/FGFR signaling pathway to oncogenesis has given rise to the advancement of a great number of therapies targeting the FGF/FGFR pathways. Though there has been no FGF/FGFR targeted therapies approved good clinical therapeutic effects in cancer treatment yet, the results from plenty of early-phase clinical trials have verified the momentous significance on targeting FGF/FGFR in the clinic. The problem of acquired resistance to targeted tyrosine kinase inhibitors gradually emerges in the clinic, and it may also restrict the clinical application of FGF/FGFR inhibitors. Several researches have indicated this phenomenon of resistance to FGF/FGFR inhibitors in cancers and expounded the possible underlying mechanism (Figure 2).

Mutation at the gatekeeper residue of FGFR is the first important mechanism of resistance to FGF/ FGFR inhibitors in kinds of cancer types (Table 2). The gatekeeper residue is located deep in the active site, which controls access of the FGFR to the hydrophobic pocket behind the ATP-binding pocket. FGFR1^V561M^ and FGFR1^N546K^ have been reported to be closely associated with the resistance of to FGFR inhibitors 62,63. The drug-resistance mechanisms are different between these two FGFR1 mutants 63. FGFR1^N546K^ mutant showed drug resistance by increasing its affinity for ATP, whereas FGFR1^V561M^ mutant showed resistance against PD173074 by reducing its affinity for the drug 63. V Chell et al revealed that FGFR3^V555M^ gatekeeper mutation is associated with resistance to FGFR inhibitors, AZD4547 and PD173074 64. Lipika Goyal et al reported three clinical patients with FGFR2 fusion-positive cholangiocarcinoma and found that FGFR2^V564F^ confers resistance to BGJ398 by causing a steric clash with BGJ398 in its FGFR2 binding pocket 65. Another research from a RNA sequencing based analysis and mouse models indicated 7 missense mutations, I567S, N568H/T, V581L, E584G, S587L, K660R and K678M, in the kinase domain of FGFR2 66. Among them, the residues I567, N568, V581, E584 and S587 are located in the ATP-binding pocket of FGFR2, the residue K660 is adjacent to the ATP-binding site, and the residue K678 is in the FGFR2 kinase activation loop 66. Further study confirmed these mutations in FGFR2 decreased the sensitivity of AZD4547 in breast cancer 66. These researches suggested that such FGFR mutations ought to be considered as clinical evaluation proceeds. Previous paper indicated that development of covalent inhibitors may conquer resistance to FGFR inhibitors induced by mutations 67. In addition, these mutations mainly occurred in the kinase domain of FGFR, and whether the mutations can mediate the resistance to several documented FGFR inhibitors, which achieved their activity via an extracellularly acting and allosteric manner, need to be further explored.

Feedback activation of survival loop caused by FGFR inhibition is another important mechanism for the resistance to FGFR inhibitors (Table 3). High-throughput proteomic approach on DMS114 (SCLC cell line) and RT112 (urothelial carcinoma cell line) cells exposed to BGJ398 indicated that increased activation of AKT and its target GSK3 may account for the acquired resistance to FGFR suppression in cancer cells 68. Another genetic screen identified that PI3K pathway could be served as a determinant of resistance to FGFR inhibitors in urothelial carcinoma 69. Further study uncovered that the elevated PI3K pathway activity may result from EGFR or Erb-B2 receptor tyrosine kinase 3 (ERBB3) reactivation induced by FGFR inhibition 69. Parallel RNA interference screens on FGFR3 mutant cancer cells also confirmed the crucial roles of EGFR played in resistance to FGFR inhibition 70. Their deeper data demonstrated that transient downregulation of MAPK signaling induced by FGFR inhibition may result in the continuous activation of EGFR via decreasing ubiquitylation 70. In endometrial cancer cell lines, loss of PTEN has been suggested as a potential mechanism of resistance to FGFR inhibition 71. Noticeably, cotreatment of FGFR2 and mTOR has a synergistic effect on the growth of endometrial cancer cell lines bearing an activating FGFR2 mutation, irrespective of PTEN status 71. In BC, overexpression of MET, inactivation of RASA1 (Ras p21 protein activator 1), and activation of drug efflux transporter ABCG2 (adenosine triphosphate binding protein G2) have been reported to be the resistance mechanism to AZD4547 66. In addition, previous research from FGFR inhibitor resistant gastric cancer cell lines indicated that EMT may be also responsible for the resistance to FGFR inhibition 72. For this situation, combined therapy may be a better solution for overcoming resistance to FGFR inhibitors.

## Conclusion

Aberrant activation of ligand dependent or independent FGF/FGFR signaling pathway makes a substantial contribution to oncotherapy resistance via promoting proliferation, survival, angiogenesis or EMT. Other non-canonical signaling mediated by FGF/FGFR, such as nuclear transition of FGFR and FGF 6, FGF/FGFR induced autophagy 73, FGF/FGFR related DNA reparation 74, may also be implicated in the resistance to oncotherapy and should be reinforced in the future study. These researches portend that combined therapy of FGF/FGFR with other oncotherapy may be a potential strategy to conquer resistance in cancers. Unfortunately, none selective FGF/FGFR inhibitors have been applied for clinic, making it difficult to further verify the clinical effectiveness of the combination strategy. Acceleration of FGF/FGFR inhibitors research is necessary and may bring pleasantly surprise for cancer treatment. It is also critical that FGFR mutant or feedback survival pathway may desensitize tumor cells to FGF/FGFR inhibitors. For reducing FGF/FGFR inhibitors resistance, covalent or extracellularly-acting inhibitors even combined therapy may be worthy of further research.

## Figures and Tables

**Table 1:** Corresponding FGF/FGFR account for resistance to therapy in cancers

Cancer types	Therapeutic method	Invovled FGF/FGFR
Lung cancer	Erlotinib	FGF13, FGFR1/2/3
	Gefitinib	FGF2/9, FGFR1/2/3
	Afatinib	FGFR1
	Trametinib	FGFR1
	Palbociclib	FGFR1
	Cisplatin	FGF2
Breast cancer	Tamoxifen	FGF1/2/4, FGFR1
	Fulvestrant	FGF1/2/4, FGFR1
	Pictilisib	FGF2
	Everolimus	FGF2
	Bevacizumab	FGF2
	Doxorubicin	FGF2, FGFR4
	Cyclophosphamide	FGFR4
Colorectal cancer	radiotherapy	FGFR4
	5-Fu	FGFR4
	Oxaliplatin	FGFR4
	5-Fu+radiotherapy	FGFR2
Gastrointestinal stromal tumor	Imatinib	FGF2, FGFR1/3
Squamous cell carcinoma	Radiotherapy	FGF2, FGFR3
	Bevacizumab	FGF2
	Paclitaxel	FGF2
	Lapatinib	FGF1/7/10
Osteosarcoma	Cisplatin	FGF2
Hepatocellular carcinoma	Gefitinib	FGF8
	Sorafenib	FGFR1
Pancreatic tumors	Antiangiogenic therapy	FGF2
Melanoma	Temozolomide	FGF2
	PLX51107	FGF2
Chronic lymphocytic leukemia	Fludarabine	FGF2
Myeloma	Dexamethasone	FGFR3
Urothelial cancer	Gemcitabine	FGFR3
	Adriamycin	FGFR3
Prostate cancer	Castration	FGF8

CDK4 inhibitor: Palbociclib; Antiestrogens: Tamoxifen, Fulvestrant; PI3K inhibitor: Pictilisib; mTOR inhibitor: Everolimus; BET inhibitor: PLX51107.

**Table 2:** Mutations induce resistance to FGFR inhibitors

FGFR	Mutated site	FGFR inhibitors
FGFR1	V561M	PD173074, dovitinib, ponatinib, BGJ-398, E3810
FGFR1	N546K	PD173074, dovitinib, ponatinib, BGJ-398
FGFR2	V564F	BGJ-398
FGFR2	I567S	AZD4547
FGFR2	N568H/T	AZD4547
FGFR2	V581L	AZD4547
FGFR2	E584G	AZD4547
FGFR2	S587L	AZD4547
FGFR2	K600R	AZD4547
FGFR2	K678M	AZD4547
FGFR3	V555M	AZD4547, PD173074

**Table 3:** Signaling pathway induces resistance to FGFR inhibitors

Cancer types	FGFR inhibitors	Involved signaling pathway
Lung cancer	BGJ398	PI3K/AKT and GSK signaling pathway
Urothelial cancer	BGJ398	PI3K/AKT and GSK signaling pathway
Urothelial cancer	AZD4547	EGFR/ERBB3-AKT signaling pathway
Bladder cancer	PD173074	EGFR signaling pathway
Endometrial cancer	Ponatinib	Loss of PTEN
Breast cancer	AZD4547	MET, inactivation of RASA1, drug-efflux
Gastric cancer	AZD4547	EMT
Gastric cancer	BGJ398	EMT
Gastric cancer	PD173074	EMT

## References

[1] Babina IS, Turner NC (2017). Advances and challenges in targeting FGFR signalling in cancer. Nat Rev Cancer.

[2] Turner N, Grose R (2010). Fibroblast growth factor signalling from development to cancer. Nat Rev Cancer.

[3] Chang J, Liu X, Wang S (2014). Prognostic value of FGFR gene amplification in patients with different types of cancer: a systematic review and meta-analysis. PLoS One.

[4] Greulich H, Pollock PM (2011). Targeting mutant fibroblast growth factor receptors in cancer. Trends Mol Med.

[5] Ahmad I, Iwata T, Leung HY (2012). Mechanisms of FGFR-mediated carcinogenesis. Biochimica et Biophysica Acta.

[6] Bryant DM, Stow JL (2005). Nuclear translocation of cell-surface recep tors: lessons from fibroblast growth factor. Traffic.

[7] Bray F, Ferlay J, Soerjomataram I (2018). Global cancer statistics 2018: GLOBOCAN estimates of incidence and mortality worldwide for 36 cancers in 185 countries. CA Cancer J Clin.

[8] da Cunha Santos G, Shepherd FA, Tsao MS (2011). EGFR mutations and lung cancer. Ann Rev Pathol.

[9] Smith BN, Bhowmick NA (2016). Role of EMT in Metastasis and Therapy Resistance. J Clin Med.

[10] Thomson S, Petti F, Sujka-Kwok I (2008). Kinase switching in mesenchymal-like non-small cell lung cancer lines contributes to EGFR inhibitor resistance through pathway redundancy. Clin Exp Metastasis.

[11] Kurimoto R, Iwasawa S, Ebata T (2016). Drug resistance originating from a TGF-β/FGF-2-driven epithelial-to-mesenchymal transition and its reversion in human lung adenocarcinoma cell lines harboring an EGFR mutation. Int J Oncol.

[12] Ware KE, Hinz TK, Kleczko E (2013). A mechanism of resistance to gefitinib mediated by cellular reprogramming and the acquisition of an FGF2-FGFR1 autocrine growth loop. Oncogenesis.

[13] Marek L, Ware KE, Fritzsche A (2009). Fibroblast Growth Factor (FGF) and FGF Receptor-Mediated Autocrine Signaling in Non-Small-Cell Lung Cancer Cells. Mol Pharmacol.

[14] Azuma K, Kawahara A, Sonoda K (2014). FGFR1 activation is an escape mechanism in human lung cancer cells resistant to afatinib, a pan-EGFR family kinase inhibitor. Oncotarget.

[15] Ware KE, Marshall ME, Heasley LR (2010). Rapidly Acquired Resistance to EGFR Tyrosine Kinase Inhibitors in NSCLC Cell Lines through De-Repression of FGFR2 and FGFR3 Expression. PLoS One.

[16] Wood K, Hensing T, Malik R (2016). Prognostic and Predictive Value in KRAS in Non-Small-Cell Lung Cancer: A Review. JAMA Oncol.

[17] Mao C, Qiu LX, Liao RY (2010). KRAS mutations and resistance to EGFR-TKIs treatment in patients with non-small cell lung cancer: a meta-analysis of 22 studies. Lung Cancer.

[18] Lee HJ, Zhuang G, Cao Y (2014). Drug Resistance via Feedback Activation of Stat3 in Oncogene-Addicted Cancer Cells. Cancer Cell.

[19] Manchado E, Weissmueller S, Morris JP 4th (2016). A combinatorial strategy for treating KRAS mutant lung cancer. Nature.

[20] Haines E, Chen T, Kommajosyula N (2018). Palbociclib resistance confers dependence on an FGFR-MAP kinase-mTOR-driven pathway in KRAS-mutant non-small cell lung cancer. Oncotarget.

[21] Pardo OE, Lesay A, Arcaro A (2003). Fibroblast Growth Factor 2-Mediated Translational Control of IAPs Blocks Mitochondrial Release of Smac/DIABLO. Mol Cell Biol.

[22] Pardo OE, Wellbrock C, Khanzada UK (2006). FGF-2 protects small cell lung cancer cells from apoptosis through a complex involving PKCε, B-Raf and S6K2. EMBO J.

[23] McLeskey SW, Zhang L, El-Ashry D (1998). Tamoxifen-resistant Fibroblast Growth Factor-transfected MCF-7 Cells Are Cross-Resistant in Vivo to the Antiestrogen IC182,780 and Two Aromatase Inhibitors. Clin Cancer Res.

[24] Manuvakhova M, Thottassery JV, Hays S (2006). Expression of the SNT-1/FRS2 phosphotyrosine binding domain inhibits activation of MAP kinase and PI3-kinase pathways and antiestrogen resistant growth induced by FGF-1 in human breast carcinoma cells. Oncogene.

[25] Thottassery JV, Sun Y, Westbrook L (2004). Prolonged Extracellular Signal-Regulated Kinase 1/2 Activation during Fibroblast Growth Factor 1- or Heregulin 1-Induced Antiestrogen-Resistant Growth of Breast Cancer Cells Is Resistant to Mitogen-Activated Protein/Extracellular Regulated Kinase Kinase Inhibitors. Cancer Res.

[26] Turner N, Pearson A, Sharpe R (2010). FGFR1 amplification drives endocrine therapy resistance and is a therapeutic target in breast cancer. Cancer Res.

[27] Shee K, Yang W, Hinds JW (2018). Therapeutically targeting tumor microenvironment- mediated drug resistance in estrogen receptor-positive breast cancer. J Exp Med.

[28] Formisano L, Stauffer KM, Young CD (2017). Association of FGFR1 with ERα\r maintains ligand-independent ER transcription and mediates resistance to estrogen deprivation in ER+ breast cancer. Clin Cancer Res.

[29] Incio J, Ligibel JA, McManus DT (2018). Obesity promotes resistance to anti-VEGF therapy in breast cancer by up-regulating IL-6 and potentially FGF-2. Sci Transl Med.

[30] Roidl A, Berger HJ, Kumar S (2009). Resistance to Chemotherapy Is Associated with Fibroblast Growth Factor Receptor 4 Up-Regulation. Clin Cancer Res.

[31] Xu M, Chen S, Yang W (2018). FGFR4 Links Glucose Metabolism and Chemotherapy Resistance in Breast Cancer. Cell Physiol Biochem.

[32] Li S, Payne S, Wang F (2015). Nuclear basic fibroblast growth factor regulates triple-negative breast cancer chemo-resistance. Breast Cancer Res.

[33] Li CF, He HL, Wang JY (2014). Fibroblast growth factor receptor 2 overexpression is predictive of poor prognosis in rectal cancer patients receiving neoadjuvant chemoradiotherapy. J Clin Pathol.

[34] Turkington RC, Longley DB, Allen WL (2014). Fibroblast growth factor receptor 4 (FGFR4): a targetable regulator of drug resistance in colorectal cancer. Cell Death Dis.

[35] Ahmed MA, Selzer E, Dörr W (2016). Fibroblast growth factor receptor 4 induced resistance to radiation therapy in colorectal cancer. Oncotarget.

[36] Kawabata M, Kawabata T, Nishibori M (2005). Role of recA/Rad51 family proteins in mammals. Acta Med Okayama.

[37] Sayed AE, Igarashi K, Watanabe-Asaka T (2017). Double strand break repair and γ-H2AX formation in erythrocytes of medaka (Oryzias latipes) after γ-irradiation. Environ Pollut.

[38] Bauer S, Duensing A, Demetri GD (2007). KIT oncogenic signalinmechanisms in imatinib-resistant gastrointestinal stromal tumor: PIkinase/AKT is a crucial survival pathway. Oncogene.

[39] Antonescu CR, Besmer P, Guo T (2005). Acquired resistance to imatinib in gastrointestinal stromal tumor occurs through secondary gene mutation. Clin Cancer Res.

[40] Javidi-Sharifi N, Traer E, Martinez J (2015). Crosstalk between KIT and FGFR3 Promotes Gastrointestinal Stromal Tumor Cell Growth and Drug Resistance. Cancer Res.

[41] Li F, Huynh H, Li X (2015). FGFR-Mediated Reactivation of MAPK Signaling Attenuates Antitumor Effects of Imatinib in Gastrointestinal Stromal Tumors. Cancer Discov.

[42] Mason JM, Morrison DJ, Basson MA (2006). Sprouty proteins: multifaceted negative-feedback regulators of receptor tyrosine kinase signaling. Trends Cell Biol.

[43] Kelly CM, Shoushtari AN, Qin LX (2019). A phase Ib study of BGJ398, a pan-FGFR kinase inhibitor in combination with imatinib in patients with advanced gastrointestinal stromal tumor. Invest New Drugs.

[44] Boichuk S, Galembikova A, Dunaev P (2018). Targeting of FGF-Signaling Re-Sensitizes Gastrointestinal Stromal Tumors (GIST) to Imatinib In Vitro and In Vivo. Molecules.

[45] Brieger J, Schroeder P, Gosepath J (2005). Vascular endothelial growth factor and basic fibroblast growth factor are released by squamous cell carcinoma cells after irradiation and increase resistance to subsequent irradiation. Int J Mol Med.

[46] Uzawa K, Ishigami T, Fushimi K (2011). Targeting fibroblast growth factor receptor 3 enhances radiosensitivity in human squamous cancer cells. Oncogene.

[47] Gyanchandani R, Ortega Alves MV (2013). A Proangiogenic Signature is Revealed in FGF-Mediated Bevacizumab Resistant Head and Neck Squamous Cell Carcinoma. Mol Cancer Res.

[48] Saito S, Morishima K, Ui T (2015). The role of HGF/MET and FGF/FGFR in fibroblast derived growth stimulation and lapatinib-resistance of esophageal squamous cell carcinoma. BMC Cancer.

[49] Fernanda Amary M, Ye H, Berisha F (2014). Fibroblastic growth factor receptor 1 amplification in osteosarcoma is associated with poor response to neo-adjuvant chemotherapy. Cancer Med.

[50] Carmo CR, Lyons-Lewis J, Seckl MJ (2011). A Novel Requirement for Janus Kinases as Mediators of Drug Resistance Induced by Fibroblast Growth Factor-2 in Human Cancer Cells. PLoS One.

[51] Pei Y, Sun X, Guo X (2017). FGF8 promotes cell proliferation and resistance to EGFR inhibitors via upregulation of EGFR in human hepatocellular carcinoma cells. Oncol Rep.

[52] Tovar V, Cornella H, Moeini A (2017). Tumour initiating cells and IGF/FGF signalling contribute to sorafenib resistance in hepatocellular carcinoma. Gut.

[53] Huynh H, Lee LY, Goh KY (2019). Infigratinib mediates vascular normalization, impairs metastasis and improves chemotherapy in hepatocellular carcinoma. Hepatology.

[54] Allen E, Walters IB, Hanahan D (2011). Brivanib, a dual FGF/VEGF inhibitor, is active both 1st and 2nd line against mouse pancreatic neuroendocrine tumors (PNET) developing adaptive/evasive resistance to VEGF inhibition. Clin Cancer Res.

[55] Casanovas O, Hicklin DJ, Bergers G (2005). Drug resistance by evasion of antiangiogenic targeting of VEGF signaling in late-stage pancreatic islet tumors. Cancer Cell.

[56] Chua V, Orloff M, Teh JL (2019). Stromal fibroblast growth factor 2 reduces the efficacy of bromodomain inhibitors in uveal melanoma. EMBO Mol Med.

[57] Fontijn D, Adema AD, Bhakat KK (2007). O^6^-Methylguanine-DNA- methyltransferase promoter demethylation is involved in basic fibroblast growth factor-induced resistance against temozolomide in human melanoma cells. Mol Cancer Ther.

[58] Pollett JB, Trudel S, Stern D (2002). Overexpression of the myeloma-associated oncogene fibroblast growth factor receptor 3 confers dexamethasone resistance. Blood.

[59] Menzel T, Rahman Z, Calleja E (1996). Elevated Intracellular Level of Basic Fibroblast Growth Factor Correlates With Stage of Chronic Lymphocytic Leukemia and Is Associated With Resistance to Fludarabine. Blood.

[60] Bluemn EG, Coleman IM, Lucas JM (2017). Androgen Receptor Pathway- Independent Prostate Cancer Is Sustained through FGF Signaling. Cancer Cell.

[61] Kikuchi A, Suzuki T, Nakazawa T (2017). ASP5878, a selective FGFR inhibitor, to treat FGFR3-dependent urothelial cancer with or without chemoresistance. Cancer Sci.

[62] Sohl CD, Ryan MR, Luo B (2015). Illuminating the Molecular Mechanisms of Tyrosine Kinase Inhibitor Resistance for the FGFR1 Gatekeeper Mutation: The Achilles' Heel of Targeted Therapy. ACS Chem Biol.

[63] Yoza K, Himeno R, Amano S (2016). Biophysical characterization of drug-resistant mutants of fibroblast growth factor receptor 1. Genes Cells.

[64] Chell V, Balmanno K, Little AS (2013). Tumour cell responses to new fibroblast growth factor receptor tyrosine kinase inhibitors and identification of a gatekeeper mutation in FGFR3 as a mechanism of acquired resistance. Oncogene.

[65] Goyal L, Saha SK, Liu LY (2017). Polyclonal secondary FGFR2 mutations drive acquired resistance to FGFR inhibition in patients with FGFR2 fusion-positive cholangiocarcinoma. Cancer Discov.

[66] Kas SM, de Ruiter J, Schut E (2018). Transcriptomics and Transposon Mutagenesis Identify Multiple Mechanisms of Resistance to the FGFR Inhibitor AZD4547. Cancer Res.

[67] Tan L, Wang J, Tanizaki J (2014). Development of covalent inhibitors that can overcome resistance to first-generation FGFR kinase inhibitors. Proc Natl Acad Sci U S A.

[68] Datta J, Damodaran S, Parks H (2017). Akt Activation Mediates Acquired Resistance to Fibroblast Growth Factor Receptor Inhibitor BGJ398. Mol Cancer Ther.

[69] Wang L, Šuštić T, Leite de Oliveira R (2017). A Functional Genetic Screen Identifies the Phosphoinositide 3-kinase Pathway as a Determinant of Resistance to Fibroblast Growth Factor Receptor Inhibitors in FGFR Mutant Urothelial Cell Carcinoma. Eur Urol.

[70] Herrera-Abreu MT, Pearson A, Campbell J (2013). Parallel RNA interference screens identify EGFR activation as an escape mechanism in FGFR3 mutant cancer. Cancer Discov.

[71] Gozgit JM, Squillace RM, Wongchenko MJ (2013). Combined targeting of FGFR2 and mTOR by ponatinib and ridaforolimus results in synergistic antitumor activity in FGFR2 mutant endometrial cancer models. Cancer Chemother Pharmacol.

[72] Grygielewicz P, Dymek B, Bujak A (2016). Epithelial-mesenchymal transition confers resistance to selective FGFR inhibitors in SNU-16 gastric cancer cells. Gastric Cancer.

[73] Cinque L, Forrester A, Bartolomeo R (2015). FGF signalling regulates bone growth through autophagy. Nature.

[74] Harfouche G, Vaigot P, Rachidi W (2010). Fibroblast Growth Factor Type 2 Signaling Is Critical for DNA Repair in Human Keratinocyte Stem Cells. Stem Cells.

